# Sustainability of Weight Loss Through Smartphone Apps: Systematic Review and Meta-analysis on Anthropometric, Metabolic, and Dietary Outcomes

**DOI:** 10.2196/40141

**Published:** 2022-09-21

**Authors:** Han Shi Jocelyn Chew, Wee Ling Koh, Janelle Shaina Hui Yi Ng, Ker Kan Tan

**Affiliations:** 1 Alice Lee Centre for Nursing Studies Yong Loo Lin School of Medicine National University of Singapore Singapore Singapore; 2 Department of Surgery Yong Loo Lin School of Medicine National University of Singapore Singapore Singapore; 3 Department of Surgery National University Hospital Singapore Singapore

**Keywords:** smartphone app, mobile app, mobile health, mHealth, eHealth, weight management, weight loss, obesity, app, diet, eating, mobile phone

## Abstract

**Background:**

Evidence on the long-term effects of weight management smartphone apps on various weight-related outcomes remains scarce.

**Objective:**

In this review, we aimed to examine the effects of smartphone apps on anthropometric, metabolic, and dietary outcomes at various time points.

**Methods:**

Articles published from database inception to March 10, 2022 were searched, from 7 databases (Embase, CINAHL, PubMed, PsycINFO, Cochrane Library, Scopus, and Web of Science) using forward and backward citation tracking. All randomized controlled trials that reported weight change as an outcome in adults with overweight and obesity were included. We performed separate meta-analyses using random effects models for weight, waist circumference, high-density lipoprotein cholesterol, low-density lipoprotein cholesterol, blood glucose level, blood pressure, and total energy intake per day. Methodological quality was assessed using the Cochrane Risk of Bias tool.

**Results:**

Based on our meta-analyses, weight loss was sustained between 3 and 12 months, with a peak of 2.18 kg at 3 months that tapered down to 1.63 kg at 12 months. We did not find significant benefits of weight loss on the secondary outcomes examined, except for a slight improvement in systolic blood pressure at 3 months. Most of the included studies covered app-based interventions that comprised of components beyond food logging, such as real-time diet and exercise self-monitoring, personalized and remote progress tracking, timely feedback provision, smart devices that synchronized activity and weight data to smartphones, and libraries of diet and physical activity ideas.

**Conclusions:**

Smartphone weight loss apps are effective in initiating and sustaining weight loss between 3 and 12 months, but their effects are minimal in their current states. Future studies could consider the various aspects of the socioecological model. Conversational and dialectic components that simulate health coaches could be useful to enhance user engagement and outcome effectiveness.

**Trial Registration:**

International Prospective Register of Systematic Reviews (PROSPERO) CRD42022329197; https://www.crd.york.ac.uk/prospero/display_record.php?RecordID=329197

## Introduction

Obesity is a metabolic disorder characterized by an excessive accumulation of fat, which is well known to increase one’s risk of cardiometabolic diseases [1], psychological stress [2], and improve overall quality of life [3]. A weight reduction of 5%-10% has been shown to lower the risk of cardiometabolic diseases by improving cardiometabolic measures such as high waist circumference, high-density lipoprotein cholesterol (HDL-C), low-density lipoprotein cholesterol (LDL-C), high blood glucose level, and high blood pressure [4]. However, weight loss through behavioral modification remains challenging because of the lack of self-regulation and motivation [5].

Common weight management strategies include lifestyle modifications (eg diet and exercise), medications, and surgery, of which the former remains the safest, most conservative, and most adopted option. Self-regulation strategies such as self-monitoring, goal setting, action planning, and relapse prevention (eg, coping planning, stress and emotion management, and dietary lapse prevention) have been shown to improve weight loss and weight loss maintenance [6]. Recent research has also reported benefits of using technology to help users to promote a state of calorie deficit through dietary self-regulation. However, the implementation of such self-regulation strategies and their long-term effects on weight loss maintenance remain unclear.

Several meta-analyses have examined the effectiveness of smartphone apps on weight loss in adults but with several limitations. In 2015, the first meta-analysis on the effectiveness of mobile phone apps reported a significant weight loss of 1.04 kg [7]. This meta-analysis was based on 12 articles searched from 3 electronic databases. Articles on people with diseases other than obesity and mobile interventions with only SMS text messaging were excluded [7]. Another study focused on the effects of mobile apps on weight loss in the Asian population and reported a small to moderate interventional effect on weight loss (Hedges *g*=−0.26) [8]. Lastly, a study on both children and adults reported a pooled interventional effect- of −1.07 kg on weight loss. However, these reviews included studies of various study designs, such as randomized controlled trials (RCTs), case-control studies, and quasi-experimental studies, which could have reduced the quality and certainty of evidence [7]. Moreover, these systematic reviews included studies on both people with normal and high BMI [7,9] and studies on people with and without diseases (eg, cardiovascular diseases) [8]. These limitations render the interventional effects on each unique population unclear.

To the best of our knowledge, none of these systematic reviews examined the effects of smartphone apps on anthropometric, metabolic, and dietary outcomes across multiple time points to examine the sustainability of interventional effects on these outcomes. Therefore, we aimed to examine the effects of smartphone apps on anthropometric, metabolic, and dietary outcomes.

## Methods

### Overview

We conducted this review according to the PRISMA (Preferred Reporting Items for Systematic Reviews and Meta-Analyses) guidelines [10] (Multimedia Appendix 1) and registered it with PROSPERO (International Prospective Register of Systematic Reviews; CRD42022329197). Two or more reviewers assessed the study selection and risk of bias (ROB). Interrater agreements were assessed using Cohen κ, where Cohen κ=0.00-0.20 indicates no agreement, Cohen κ=0.21-0.39 indicates weak agreement, Cohen κ=0.40-0.59 indicates minimal agreement, Cohen κ=0.60-0.79 indicates moderate agreement, Cohen κ=0.80-0.90 indicates strong agreement, and Cohen κ>0.90 indicates almost perfect agreement [11].

### Search Strategy

We conducted a systematic search through 7 databases (CINAHL, PsycINFO, PubMed, Scopus, Cochrane Library, and Web of Science) for relevant articles published from database inception to March 10, 2022. Keywords and Medical Subject Headings terms were first searched through PubMed and Embase to permute more keywords, namely, *smartphone application*, *phone application*, *mobile*, *app*, *m-health*, *mHealth*, *obesity*, *overweight*, *body weight*, *weight loss*, *weight reduction*, and *weight management* (Multimedia Appendix 2 provides the detailed database keyword search). Citations were screened using the EndNote software (Clarivate). Full-text articles were independently screened by 3 reviewers (HSJC, JSHYN, and WLK).

### Study Selection

Titles and abstracts were first screened by HSJC according to the eligibility criteria crafted using the population, intervention, comparison condition, outcomes, and study design framework. Full-text articles were screened independently by HSJC, JSHYN, and WLK. Discrepancies were resolved among the 3 reviewers. Articles were included if they (1) were about people with high BMI (≥25 kg/m^2^ for Western populations and ≥23 kg/m^2^ for Asian populations; (2) examined the effectiveness of a smartphone app; (3) examined interventional effect on at least weight loss as an outcome; (4) reported outcomes beyond baseline and after intervention; and (5) had an RCT study design. Articles were excluded if they (1) focused on people with medical conditions (excluding overweight and obesity), (2) were not empirical RCTs (eg, pilot studies and secondary analyses of RCTs, in which case the original RCT was retrieved), (3) examined smartphone apps that only provided SMS text messaging interventions such as SMS text message reminders, and (4) did not have an English version of the manuscript.

### Data Extraction

Data extraction was performed independently by HSJC and WLK according to an Excel spreadsheet template for information on the following criteria: authors, year of publication, country, sample size, sample characteristics, the cutoff BMI score for having overweight (kg/m^2^), mean age, percentage of male participants, socioeconomic status, educational level, weight measure, baseline weight, baseline BMI, app components, control condition, intervention duration, follow-up time points, attrition rate by the time of analysis, any significant baseline differences between the participants retained and those lost to follow-up, missing data management, whether the protocol was registered, whether the study was funded, and study outcomes in terms of mean (SD), mean (SE), or mean difference (MD; 95% CIs). For data extraction from 3-armed RCTs, pooled intervention outcome data were used if both intervention arms comprised different apps. Otherwise, only the intervention arm with a smartphone app was extracted. For studies that reported a separate intervention arm with additional nonapp components, data were not extracted.

### Methodological Quality

The Cochrane ROB Tool was used to rate the articles’ methodological quality as low, unclear, and high ROB based on 6 domains, namely random sequence generation, allocation concealment, blinding of participants and personnel, blinding of outcome assessment, outcome data completeness, and selective reporting [12]. Ratings were performed independently by JSHYN and WLK and discrepancies were resolved through discussions with HSJC. We planned to assess the certainty of evidence using the Grading of Recommendations, Assessment, Development, and Evaluation approach but decided not to because of the limited number of studies available and a relatively high ROB, which would not have allowed us to derive a meaningful conclusion on the certainty of evidence.

### Data Analysis

Sample variance estimates reported as SEs and CIs were converted to SD. The unit kJ/day was converted to kcal/day and lb was converted to kg. Weight, waist circumference, blood glucose, total energy intake, and blood pressure effect sizes were estimated using weighted MDs (WMDs), whereas HDL-C and LDL-C were estimated using standardized MDs (SMDs). SMDs were adjusted by using Hedges *g* to account for the small number of studies included in the meta-analyses. Some studies reported results from multiple time points beyond 6 months (ie, 9, 18, and 24 months). Therefore, results from the most reported time points were used for the meta-analyses to prevent bias arising from repeated measures. All meta-analyses were conducted using random effects models with generic inverse variance and adjusted using the Hartung-Knapp-Sidik-Jonkman method instead of the commonly used DerSimonian-Laird method, as it has been shown to produce fewer type I errors, especially for analyses with a small number of studies [13]. Between-study heterogeneity was estimated using the τ^2^ statistic and quantified using the *I*^2^ statistic, where 25%, 50%, and 75% indicate a small, moderate, and large degree of heterogeneity, respectively [14]. Sensitivity analyses were performed to assess the individual effects of each study on the pooled effect size estimates, and publication bias was assessed using funnel plots and the Egger test, when possible. All statistical analyses were performed using R (version 4.1.3) [15].

## Results

### Overview

**Figure 1:**
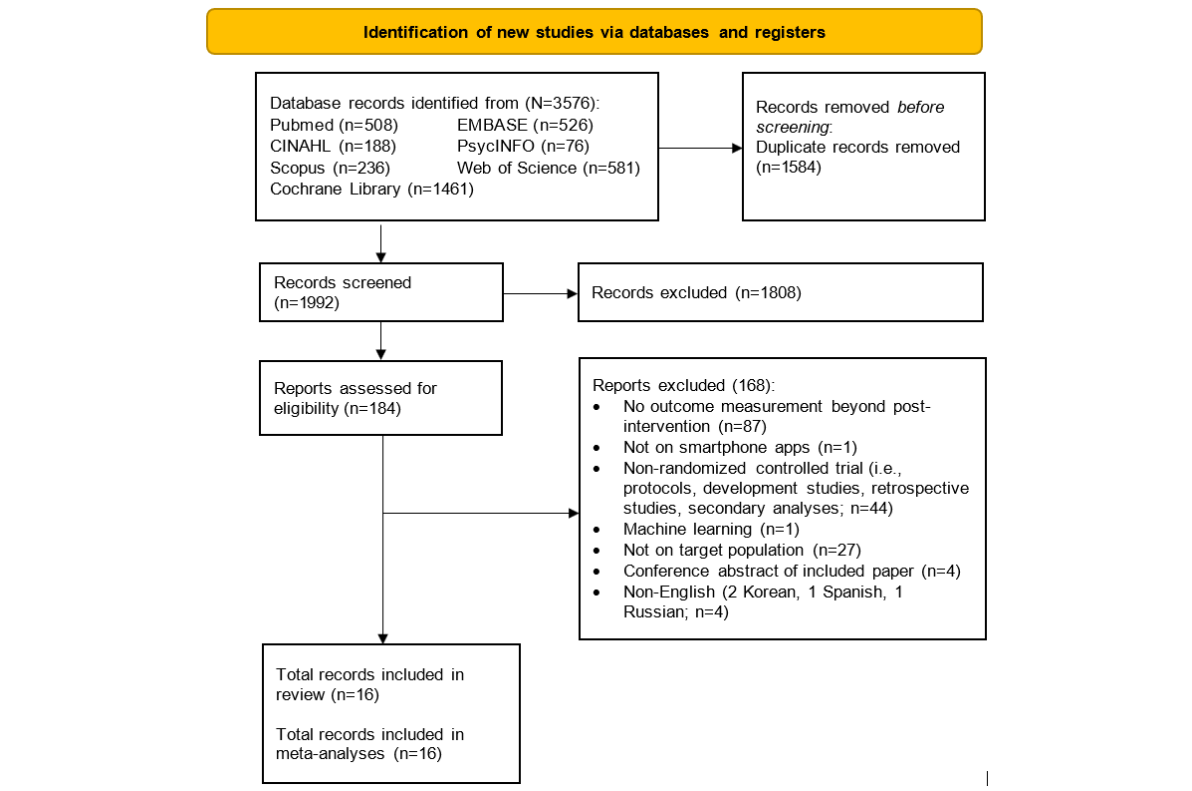
PRISMA (Preferred Reporting Items for Systematic Reviews and Meta-Analyses) flowchart.

Of the 3576 articles retrieved from our systematic database search, 1584 (44.3%) duplicate articles were removed, resulting in 1992 (55.7%) titles and abstracts screened for eligibility. After excluding 90.8% (1808/1992) of articles based on the title and abstract screening, 9.2% (184/1992) full texts were assessed, of which 168 (91.3%) articles were excluded for reasons shown in Figure 1. We also searched through the references of the included articles, but no additional articles fulfilled the inclusion criteria. A total of 16 articles were included in the review, with 68 unique effect sizes included in the meta-analyses. The interrater agreement statistics for the inclusion of the article and overall ROB were Cohen κ=0.77; *P<*.001 and Cohen κ=0.71; *P=*.003, respectively, indicating moderate agreements.

### Study Characteristics

The 16 articles represented 2870 participants with overweight or obesity, with mean ages ranging from 22.7 years to 70.1 years, mean body weight ranging from 70.6 kg to 114.1 kg (2 studies did not report data on weight [16,17]), mean BMI ranging from 27.5 kg/m^2^ to 36.2 kg/m^2^, and the proportion of male participants ranging from 9.3% to 100%. Most studies (12/16, 75%) were conducted in the United States, except for 1 each from the United Kingdom [18], Australia [19], Japan [20], and China [21]. The attrition rates ranged from 0.5% to 46.6%. A total of 69% (11/16) of articles reported data that reflected the sample’s socioeconomic status, and 81% (13/16) of articles reported data on the sample’s education level. More details on the study characteristics are shown in Table 1, and additional information on socioeconomic status, education level, presence of group differences between participants retained and dropped out, protocol registration, and funding is shown in Multimedia Appendix 3 [16-31]. Most included studies (12/16, 75%) covered app-based interventions that were beyond purely food logging, such as real-time self-monitoring of diet and exercise, regular messages tailored according to user progress, timely feedback, smart devices that synchronized activity and weight data to smartphones, personalized progress reports, libraries of diet and physical activity ideas, and remote progress monitoring. Of these 16 articles, 4 (25%) included control conditions that provided app-based food logging [17,22-24]. The intervention duration ranged from 12 weeks to 24 months and the follow-up time points ranged from 8 weeks to 24 months. The intervention characteristics for each article are detailed in Multimedia Appendix 4 [16-31]. Most articles (10/16, 62%) were rated as having an unclear ROB, and 38% (6/16) of articles were rated as having a high ROB (Multimedia Appendix 5 [16-31]). Moreover, 38% (6/16) of articles were rated as having a high ROB for performance bias because of the difficulties in blinding both the interventionists and participants, which is common in such behavior-change studies (Multimedia Appendix 5 [16-31]). Owing to the varying outcome measurement time points, results were analyzed at <3 months, 3 months, 6 months, and >6 months whenever possible.

**Table 1:** Characteristics of the 16 included randomized controlled trial articles^a^.

Study	Trial arms (n); country	Sample size (n)	Age (years), mean	Proportion of males, %	Baseline weight (kg), mean; baseline BMI (kg/m^2^), mean	Attrition rate^b^, %; presence of group differences^c^	Missing data management; protocol registration; funding	Reported on SES^d^; educational level
Carter et al [18], 2013	3; United Kingdom	128	41.9	22.7	96.9; 34.2	38.3; yes	ITT^e^; yes; yes	Yes; yes
Duncan [19], 2020	3; Australia	116	44.5	29.3	90.7; 31.7	46.6; yes	ITT; yes; yes	Yes; yes
Dunn et al [17], 2019	2; United States	43	42.4	9.3	NS^f^; 34.5	30; NS	ITT; yes; yes	Yes; yes
Eisenhauer et al [22], 2021	2; United States	80	54.2	100	114.1; 35.6	7.5; NS	NS; yes; yes	Yes; yes
Falkenhain et al [23], 2021	2; United States	155	41	29	94.4; 33.5	25.2; nil	ITT; yes; yes	Yes; yes
Godino et al [30], 2016	2; United States	404	22.7	29.7	80.7; 29	15.6; nil	ITT; yes; yes	Yes; yes
Johnston et al [26], 2013	2; United States	292	46.5	10.2	90.1; 33	12; nil	ITT; no; yes	Yes; yes
Kurtzman et al [29], 2018	3; United States	196	41.4	14.3	102.5; 36.2	4.5; NS	ITT; yes; yes	Yes; yes
Martin et al [25], 2015	2; United States	40	44.4	17.5	80.3; 29.8	5.0; NS	ITT; yes; yes	NS; NS
Patel et al [24], 2019	3; United States	105	42.7	16	89.6; 31.9	26.7; NS	ITT; yes; yes	Yes; yes
Rosas et al [31], 2020	2; United States	192	50.2	38.2	87.1; 32.4	0.5; NS	PP^g^; yes; yes	Yes; yes
Ross et al [27], 2016	3; United States	80	51.1	13.8	89.3; 33	10; NS	ITT; yes; yes	NS; NS
Spring et al [28], 2017	3; United States	96	39.3	15.6	94.8; 34.6	13.5; yes	ITT; yes; yes	NS; Yes
Tanaka et al [20], 2018	2; Japan	112	46.3	99.1	83.2; 28	27.7; NS	ITT; yes; yes	NS; NS
Turner-McGrievy et al [16], 2017	2; United States	81	48.1	17.3	NS; 33.4	25; nil	ITT; yes; yes	Yes; yes
Zhou et al [21], 2021	3; China	750	70.1	46.1	70.6; 27.5	14.4; NS	NS; yes; yes	NS; Yes

^a^Details of the group differences, protocol registration, funding, reports on socioeconomic status, and educational levels are presented in Multimedia Appendix 3 [16-31].

^b^Attrition rate by last outcome measurement time point.

^c^Group differences between participants who were retained and dropped out.

^d^SES: socioeconomic status.

^e^ITT: intention-to-treat.

^f^NS: not specified.

^g^PP: per-protocol analysis.

### Weight Loss

**Figure 2:**
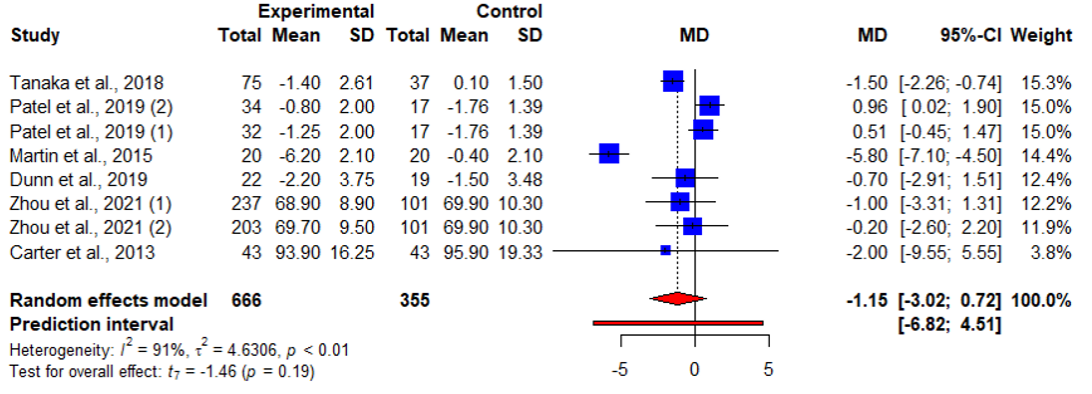
An illustration of the summary statistics of the intervention and control groups in each study included in the meta-analysis on the effect of smartphone weight loss apps on weight loss before 3 months. MD: mean difference.

All 16 articles reported results on weight change. A total of 38% (6/16) of articles reported results of weight change at 3 months, of which 4 (67%) reported significant weight loss [18,20,21,25] and 2 (33%) reported otherwise [17,24]. Of these 4 articles, 2 (50%) reported results of 2 intervention arms with consistent findings on interventional effects on weight change and were analyzed as 4 studies in the meta-analysis [21,24]. The pooled WMD suggested a nonsignificant interventional effect on weight loss at <3 months (sample size, n=8; WMD=−1.15, 95% CI −3.02 to −0.72; *P=*.19; *I*^2^=91.3%; Figure 2 and Table 2).

**Figure 3:**
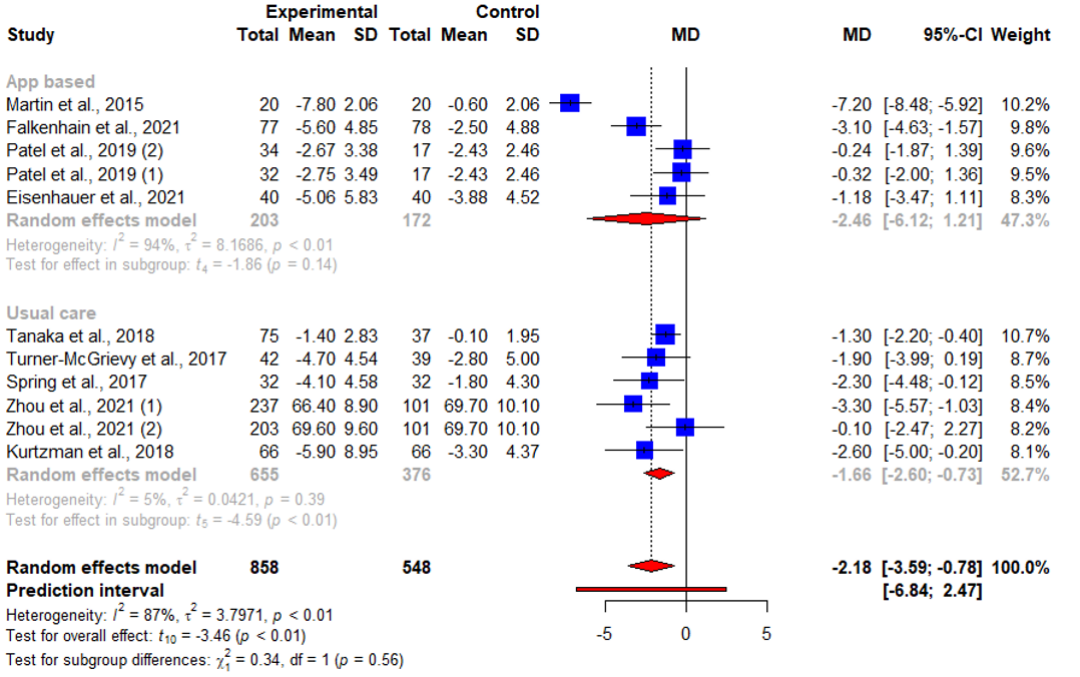
An illustration of the summary statistics of the intervention and control groups in each study included in the meta-analysis on the effect of smartphone weight loss apps on weight loss at 3 months. The illustration also shows the subgroup analysis of the studies based on whether the control group received an app-based intervention. MD: mean difference.

**Figure 4:**
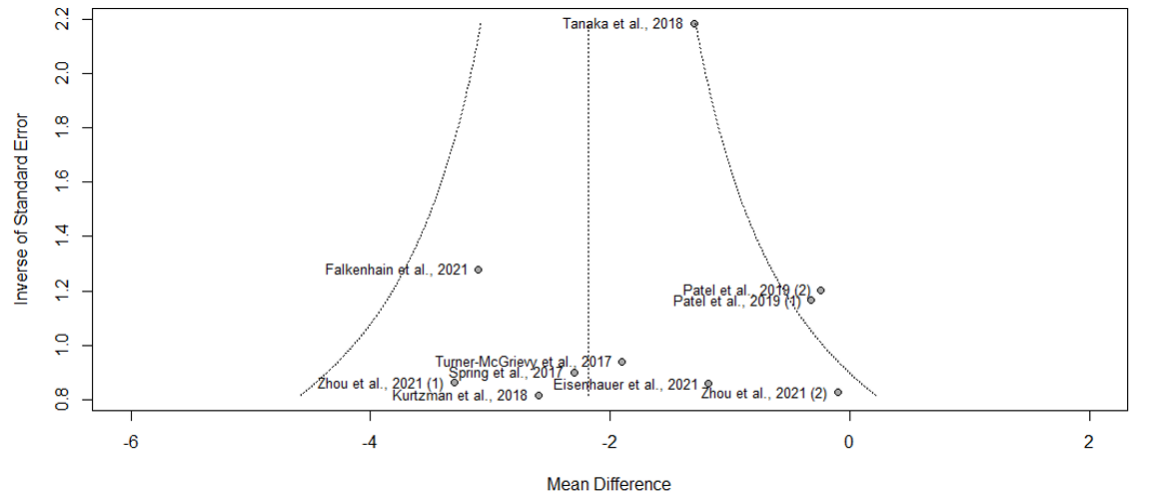
Funnel plot of symmetry for the included studies that reported the effects of smartphone weight loss apps on weight loss at 3 months.

Of the 16 articles, 11 (69%) reported results of weight change at 3 months, of which 8 (50%) reported significant weight loss [16,20,21,23,25-28] and 3 (19%) reported otherwise [22,24,29]. The pooled WMD for weight loss suggested statistically significant interventional effects at 3 months (n=11; WMD=−2.18, 95% CI −3.59 to −0.78; *P=*.006; *I*^2^=87.3%). A subgroup analysis was also conducted, in which the heterogeneity between studies was not attributed to whether the respective control groups received an app-based intervention (*Q*^2^_1_=0.34; *P=*.56; Figure 3 and Table 2). No publication bias was detected based on the symmetry of the funnel plot and the Egger test (0.85; *t*=0.31; *P=*.76; Figure 4).

**Figure 5:**
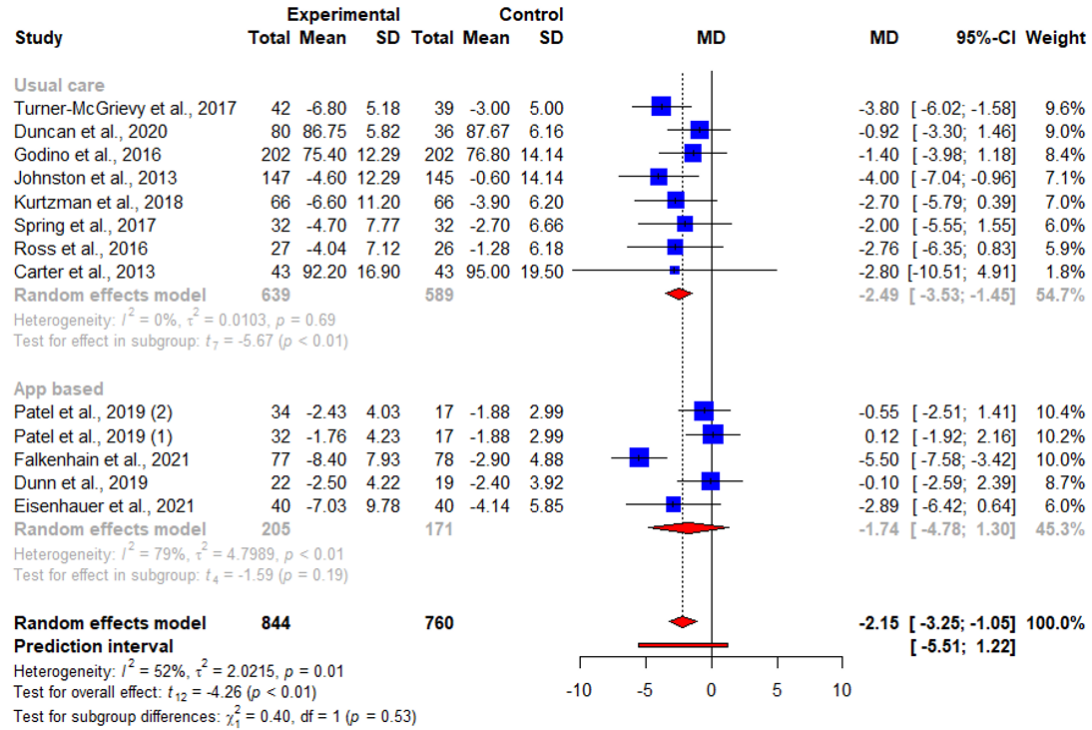
An illustration of the summary statistics of the intervention and control groups in each study included in the meta-analysis on the effect of smartphone weight loss apps on weight loss at 6 months. The illustration also shows the subgroup analysis of the studies based on whether the control group received an app-based intervention. MD: mean difference.

**Figure 6:**
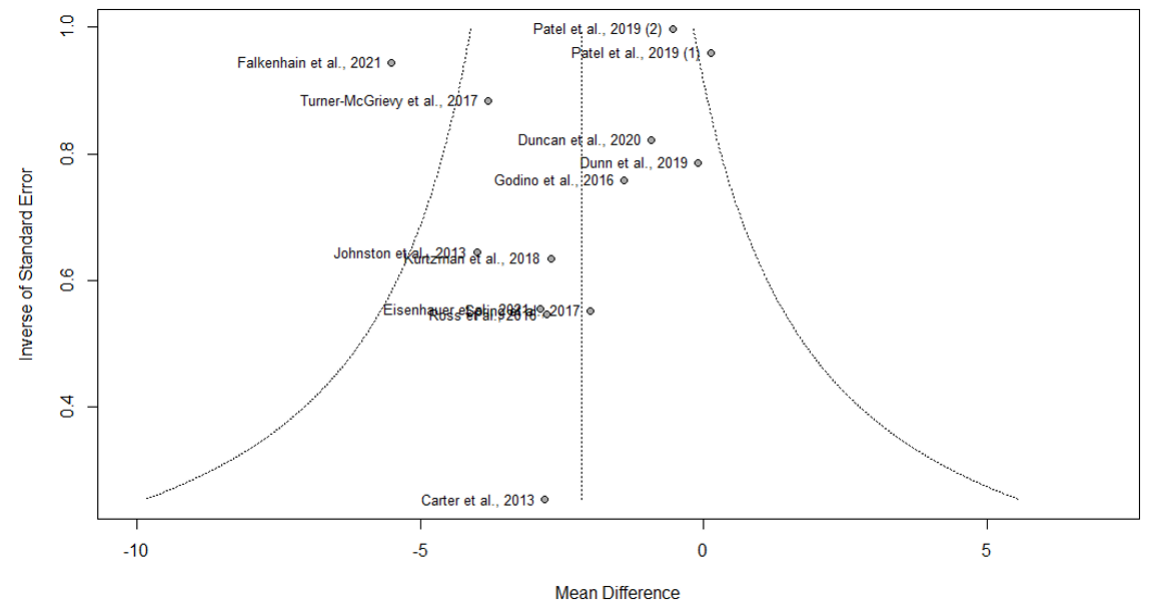
Funnel plot of symmetry for the included studies that reported the effects of smartphone weight loss apps on weight loss at 6 months.

A total of 75% (12/16) of articles reported results of weight change at 6 months, of which 7 (44%) reported significant weight loss [16,18,22,23,26-28], and 5 (31%) reported otherwise [17,19,24,29,30]. The pooled WMD for weight loss suggested statistically significant interventional effects at 6 months (n=13; WMD=−2.15, 95% CI −3.25 to −1.05; *t*=−4.26; *P=*.001; *I*^2^=52.4%; Figure 5 and Table 2). A subgroup analysis was also conducted, in which the heterogeneity between studies was not attributed to whether the respective control groups received an app-based intervention (*Q*=0.40; *P=*.53; Figure 5 and Table 2). No publication bias was detected based on the symmetry of the funnel plot and the Egger test (−0.74; *P=*.63; Figure 6).

**Figure 7:**
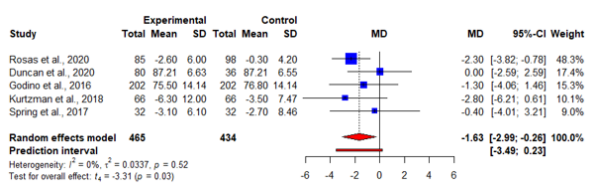
An illustration of the summary statistics of the intervention and control groups in each study included in the meta-analysis on the effect of smartphone weight loss apps on weight loss at 9 to 12 months. MD: mean difference.

In addition, 25% (4/16) of articles reported results of weight change at 12 months, of which 2 (50%) reported significant weight loss [30,31], and 2 (50%) reported otherwise [19,28]. Interventional effect was assessed at 9 months [29], 18 months [30], and 24 months [31]; only 6% (1/16) of articles reported significant weight loss at both 18 and 24 months [30]. The pooled WMD for weight loss suggested statistically significant interventional effects at 9 to 12 months (n=5; WMD=−1.63, 95% CI −2.99 to −0.26; *P=*.03; *I*^2^=0%; Figure 7 and Table 2).

**Table 2:** A summary of meta-analyses results on each outcome at each time point analyzed.

Time points		Sample size (n)	MD^a^ or SMD^b^ (95% CI)			*t* value		*P* value				*τ^2^* statistic		*I*^2^ (%)	
**Weight (kg)**															
	<3 months	8	−1.15 (−3.02 to 0.72)		−1.46				.19		4.63				91.3
	3 months	11	−2.18 (−3.59 to −0.78)		−3.46				.006^c^		3.80				87.3
	6 months	13	−2.15 (−3.25 to −1.05)		−4.26				.001^d^		2.02				52.4
	9-12 months	5	−1.63 (−2.99 to −0.26)		−3.31				.03^d^		0.03				0
**Waist circumference (cm)**															
	<3 months	4	−2.30 (−6.98 to −2.38)		−1.57				.22		6.92				81.7
	3 months	4	−3.85 (−9.31 to 1.60)		−2.25				.11		10.28				88.7
	6 months	2	−0.92 (−3.88 to 2.04)		−3.94				.16		0				0
	12 months	3	−1.19 (−3.80, to 1.43)		−1.95				.19		0				0
**HDL-C^e^**															
	3 months	2	0.01 (−0.15 to 0.17)^b^		0.80				.57		0				0
**LDL-C^f^**															
	3 months	2	−0.06 (−1.31 to 1.44)^b^		0.58				.66		0				0
**Glycated hemoglobin (HbA_1c_; %)**															
	3-6 months	3	−0.22 (−1.03 to −0.6)		−1.14				.37		0.07				67.1
**Total energy intake per day^g^**															
	6-12 months	3	−86.2 (−494.53 to 322.12)		−0.91				.46		11,382				23.9
**Systolic blood pressure (mm Hg)**															
	3 months	3		−4.67 (−5.95 to −3.40)			−15.8			.004^d^			0		0
	6 months	2		−0.28 (−15.6 to 15.03)			−0.23			.85			1.10		21
**Diastolic blood pressure (mm Hg)**															
	3 months	3		−2.88 (−8.37 to 2.62)			−2.25			.15			3.51		68.3
	6 months	2		−0.65 (−1.56 to 0.26^c^)			−9.06			.07			0		0

^a^MD: mean difference.

^b^SMD: standardized mean difference (adjusted with Hedges *g*).

^c^*P*<.05.

^d^*P*<.01.

^e^HDL-C: high-density lipoprotein cholesterol.

^f^LDL-C: low-density lipoprotein cholesterol.

^g^kJ converted to kcal.

### Waist Circumference

**Figure 8:**
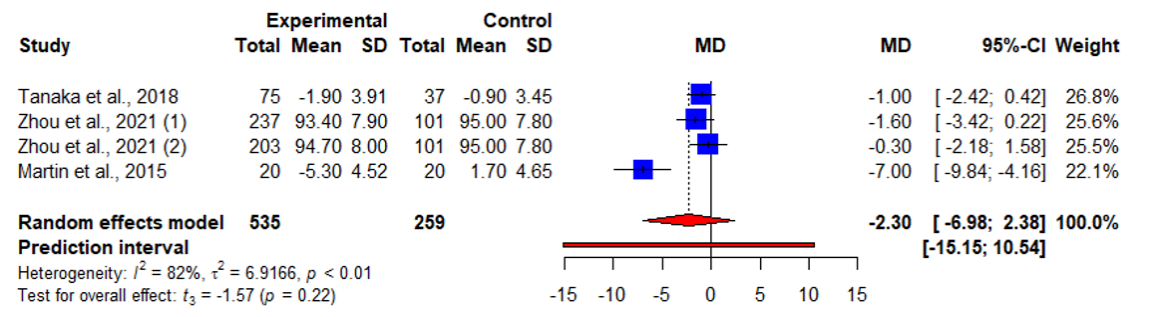
An illustration of the summary statistics of the intervention and control groups in each study included in the meta-analysis on the effect of smartphone weight loss apps on waist circumference before 3 months. MD: mean difference.

**Figure 9:**
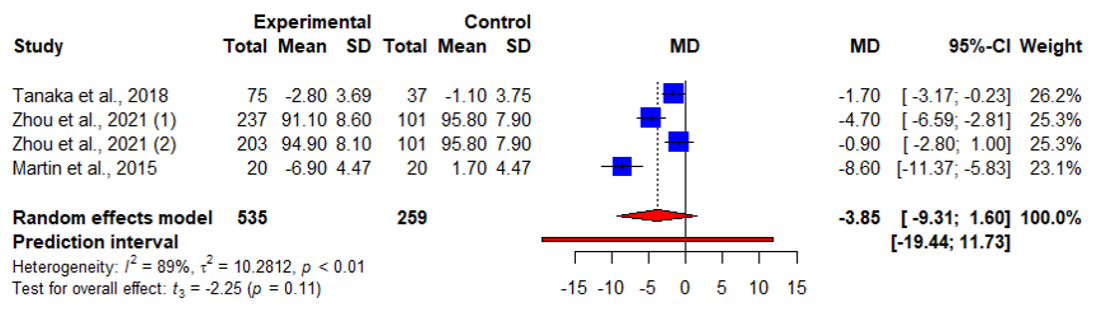
An illustration of the summary statistics of the intervention and control groups in each study included in the meta-analysis on the effect of smartphone weight loss apps on waist circumference at 3 months. MD: mean difference.

A total of 31% (5/16) of the articles measured interventional effects on waist circumference [19-21,25,30]. Of these 5 articles, 2 (40%) [21,25] reported a significant reduction in waist circumference at 3 months, whereas 1 (20%) reported otherwise [20]. Significant reductions in waist circumference were reported at 3 months [20,21,25] and 6 months [19,30], whereas different results were reported at 12 to 24 months [19,30]. No significant interventional effect was found on waist circumference beyond 4 months. No significant interventional effect was found through our meta-analyses for waist circumference before 3 months (Figure 8) and at 3 months (Figure 9), 6 months, and 12 months (Table 2).

### Calorie Intake

A total of 25% (4/16) of articles measured interventional effects on waist circumference [16,19,23,31]. Of these 4 articles, 1 (25%) article reported a significant interventional effect on calorie intake per day at 3 and 6 months [16], whereas another article (1/4, 25%) reported otherwise [23]. In addition, 50% (2/4) of articles reported a significant reduction in calorie intake per day [19,23], and 50% (2/4) of articles reported no significant result at 12 months [19,31]. No significant interventional effect was found through our meta-analyses for total calorie intake per day between 6 and 12 months (Table 2).

### HDL-C, LDL-C, and HbA1c Levels

A total of 13% (2/16) of articles reported results on HDL-C and LDL-C, in which no significant change was reported at various time points [20,23]. In total, 19% (3/16) of articles reported results on HbA_1c_, of which 13% (2/16) of articles reported a significant reduction at 3 months [20,23], and 13% (2/16) of articles reported results at 6 months but with contrasting findings [19,23]. According to our meta-analyses, no significant interventional effect was found for HDL-C, LDL-C, and HbA_1c_ levels between 3 and 6 months (Table 2).

### Blood Pressure

A total of 19% (3/16) of articles reported results on blood pressure at various time points. A significant interventional effect was found for systolic blood pressure at 3 months (Cohen κ=3; WMD=−4.67, 95% CI −5.95 to −3.40; *t*=−15.8; *P=*.004; *I*^2^=0%) but not at 6 months (Cohen κ=2; WMD=−0.28, 95% CI −15.6 to −15.03; *t*=−15.8; *P=*.004; *I*^2^=0%). No significant interventional effect was found for diastolic blood pressure at 3 and 6 months (Table 2).

A summary of the outcomes reported in each study is reported in Multimedia Appendix 6 [16-31].

## Discussion

### Principal Findings

To the best of our knowledge, this is the first systematic review and meta-analysis that analyzed the effects of smartphone weight loss apps on weight loss and other anthropometric, metabolic, and dietary outcomes across various time points. Most articles reported results on weight loss at 3 and 6 months, but few reported findings on other anthropometric, metabolic, and dietary outcomes. Based on narrative syntheses and meta-analyses of evidence from the included studies, the findings showed that the use of smartphone apps for weight loss was generally minimal and unsustainable.

Based on our meta-analyses, weight loss was sustained between 3 and 12 months, with a peak of −2.18 kg at 3 months that tapered down with time to −1.63 at 12 months. This finding is similar to a prior systematic review that reported a significant weight loss of −1.99 kg and −2.8 kg at 3 and 6 months, respectively, in a population with and without overweight or obesity [32]. The slight difference in the time of peak weight loss could be related to the additional nonmobile elements implemented alongside smartphone apps, such as health coaching, which is well established to facilitate weight loss. However, weight loss of this magnitude may not be sufficient to reach a clinically significant reduction in cardiometabolic disease risk, assuming that a 5% decrease in weight (generally accepted rule-of-thumb for a clinically meaningful weight loss) for someone with a weight of 70.6 kg (lower value of the mean age range) translates to a weight loss of 3.53 kg [33]. Moreover, the actual effectiveness of such apps could be even lower considering that participants who stay throughout the interventions have a certain level of motivation to lose weight, potentially augmenting the results. Conversely, recent studies have shown improvements in outcomes such as the risk of developing type 2 diabetes, glucose tolerance, blood pressure, and triglycerides, even with a lower weight loss of ≥2% [34]. Nevertheless, the weight loss derived from using smartphones remains limited in their current state and may only be useful for people with slight overweight. Moreover, most results were derived from a Western population, who may have different preferences, engagement, and metabolic responses as compared to an Eastern population. Future studies may consider population characteristics in their app development.

The limited weight loss observed could be related to the behavioral components included in the weight loss apps examined, as 1 study reported that some form of an intensive health coaching alongside the use of smartphone apps improved weight loss [32]. This could be because of the immaturity of app-based coaches in matching up to the competencies of real-life health coaches in terms of relatability, usability, and trust [35]. However, another study reported weak associations between behavioral components and the usability and effectiveness of smartphone apps for weight loss, suggesting the unclear role of behavioral components in similar apps [36]. Given the established effectiveness of health coaching on weight loss, future studies could explore the integration of simulated health coaches—potentially the use of conversational agents to enhance the acceptability and engagement of similar apps while reducing the manpower needed [35].

Comparing the studies that reported mixed findings on weight loss at <3 months, the significant weight loss reported in the 4 studies could be attributed to the inclusion of personalized messages delivered by the app, dieticians, or coaches [18,20,21,25], which were not present in the remaining 2 studies that only included the food logging function and reported nonsignificant findings [17,24]. This is consistent with previous studies that reported higher engagement with, preference for, and weight loss outcomes in eHealth programs that included personalized recommendations [37]. Moreover, calorie counting has been described as troublesome and dislikable by users who prefer to have more motivational aspects in weight loss apps [38]. Therefore, future studies could consider replacing manual food logging with more intuitive and automatic methods. For example, food logging can be supported by food image recognition technology, self-directed goal setting, and progress monitoring, similar to most smartphone weight loss apps, and can be personalized and calibrated using medical and constantly updated lifestyle history [39].

By contrast, our meta-analysis results suggest significant effects of smartphone weight loss apps on weight loss from 3 to 12 months. This is consistent with existing systematic reviews [7-9], although the time points at which the analyzed data were retrieved were unclear. However, both our narrative synthesis and meta-analysis did not show the benefits of weight loss on secondary outcomes, except for a slight improvement in systolic blood pressure at 3 months. This is in contrast with previous systematic reviews on studies with follow-up time points of ≥2 years. With every reduction of 10 kg in body weight, a reduction of 5% to 10% in cholesterol levels has been reported alongside significant improvements in HDL-C and LDL-C levels [40]. Similarly, for every reduction of 10 kg in body weight, a reduction of 6.0 mm Hg and 4.6 mm Hg in systolic and diastolic blood pressure levels has been reported [41]. Weight loss has also been correlated with a reduction in fasting blood glucose levels in both diabetic and nondiabetic populations [42]. Weight loss without a change in metabolic outcomes defeats the purpose of weight loss, which should be to reduce the risk of noncommunicable lifestyle diseases (eg, coronary artery diseases and diabetes) through an improvement in metabolic outcomes. However, these discrepancies are more likely because of the small number of studies included in the meta-analyses of the secondary outcomes at various time points, which could have caused the analyses to be underpowered in detecting true effects, if present. Therefore, any inferences made based on the results of the secondary outcomes should be made with discretion. Moreover, compared with prior studies, a weight loss as large as 10 kg may be required for significant improvements in cholesterol and blood pressure to be detected. Future studies should consider examining a set of standard weight-related outcomes, such as the 7 outcomes examined in this study, over multiple time points to elucidate clearer findings.

Finally, our findings did not suggest a significant reduction in total calorie intake per day, potentially because of the small sample size. However, similar findings were reported in a large RCT that included patients with existing medical conditions [43]. The use of a smartphone app together with a smart activity band resulted in greater weight loss 12 months into the program, where both the intervention and control groups were found to have decreased their calorie intake comparably. This could be because of an increase in the duration of light physical activity, which could also explain our findings on interventional effectiveness on weight loss but not calorie intake.

In this age of widespread smartphone penetration and social media influence, people with overweight and obesity are often exposed to fad diets (eg, ketogenic diet, intermittent fasting, and Atkins diet) [44], and popular physical activities such as high-intensity interval training, cardio training, and resistance training. However, studies have shown limited effects and potential health issues (eg, malnutrition, dehydration, and acute injuries) with such diets [45] and unsupervised intensive physical activities [46]. As weight management is an integral aspect of population health, we should not be typecasting or identifying people with overweight and obesity for weight management but empowering people with the skills and resources to manage their weight independently. This is especially because of the global weight gain observed during the COVID-19 pandemic [47], which could be associated with varying levels of personal motivation [5], peer influence [48], and self-regulation [49]. Although many purchased home gym equipment during this period, the sustained use of such equipment without peer influence or health coaching may not have been high.

### Recommendations

Given the limited effectiveness of smartphone apps in their current state, future studies could consider the following recommendations. Further apps or app-related programs may consider the incorporation of the socioecological model to include more factors within the complexities of overweight and obesity [50]. These include food production, societal influences, food consumption, biology, individual psychology, individual activity, and activity environment [51]. Future apps may also consider including the component of health coaching as a continual source of motivation, discipline, and guidance. This could be in the form of a human coach, conversational agent, or other interactive embodiment that simulates a human coach [52].

### Strengths and Limitations

Each step of the systematic review process, including the study selection, data extraction, and methodological assessment, was independently performed by at least two reviewers. We also reported our findings in terms of MDs instead of SMDs to provide more clinically relevant metrics on the effects of smartphone apps on weight loss. However, there were some limitations to this study. First, no gray literature was searched, which could have precluded some relevant articles, but it ensured the rigor of this study by excluding non–peer-reviewed articles. Second, the sample size of articles included in the meta-analyses was small, rendering any conclusions prone to inaccuracies and biases. Therefore, discretion is needed when readers draw inferences and conclusions based on our findings. Third, the heterogeneity between the studies included in each outcome meta-analysis was high, ranging from 21% to 91.3%. This suggests a certain degree of inaccuracy in our conclusions. Finally, most included studies were rated as having an unclear or high ROB, indicating the presence of an inherent ROB in our findings. Nevertheless, we appended additional sociodemographic information such as the socioeconomic and educational profile of each included study for readers to make better judgments of the findings presented in this paper.

### Conclusions

The use of smartphone weight loss apps at the current stage may not be sufficient to produce clinically meaningful health outcomes. Future studies could consider tackling more influencing factors of weight management at every level of the socioecological model to empower population weight management. Future studies could consider including conversational and dialectic component that simulates a health coach and provides personalized progress monitoring and feedback to enhance user engagement and outcome effectiveness.

## Appendix

Multimedia Appendix 1 PRISMA (Preferred Reporting Items for Systematic Reviews and Meta-Analyses) 2020 checklist.

Multimedia Appendix 2 Search strategy.

Multimedia Appendix 3 Extended study characteristics of the 16 included articles.

Multimedia Appendix 4 Intervention characteristics of the 16 included articles.

Multimedia Appendix 5 Methodological quality assessment of 16 included articles using the Cochrane Risk of Bias tool.

Multimedia Appendix 6 Summary of the outcomes reported in each study.

## Abbreviations

HDL-C: high-density lipoprotein cholesterol
LDL-C: low-density lipoprotein cholesterol
MD: mean difference
PRISMA: Preferred Reporting Items for Systematic Reviews and Meta-Analyses
PROSPERO: International Prospective Register of Systematic Reviews
RCT: randomized controlled trial
ROB: risk of bias
SMD: standardized mean difference
WMD: weighted mean difference
