# Supplementary material for: Sustainability of Weight Loss Through Smartphone Apps: Systematic Review and Meta-analysis on Anthropometric, Metabolic, and Dietary Outcomes
Source: J Med Internet Res. 2022 Sep 21;24(9):e40141. doi: 10.2196/40141 (PMC9536524; doi:10.2196/40141)
Supplement: Multimedia Appendix 2 [file jmir_v24i9e40141_app2.docx]

**APPENDIX 2** Search strategy

| Database | Index and keyword terms | Results |
| --- | --- | --- |
| *PubMed* | ((smartphone application[Title/Abstract] OR phone application[Title/Abstract] OR mobile application[Title/Abstract] OR app[Title/Abstract] OR m-health[Title/Abstract] OR mhealth[Title/Abstract]) AND (Obesity[Title/Abstract] OR overweight[Title/Abstract] OR body weight[Title/Abstract] OR weight loss[Title/Abstract] OR weight reduction[Title/Abstract] OR weight management[Title/Abstract])) AND (Randomized[Title/Abstract] OR randomised[Title/Abstract] OR randomly[Title/Abstract] OR random[Title/Abstract] OR control group[Title/Abstract]) | 508 |
| *EMBASE* | ('smartphone application':ab,ti OR 'phone application':ab,ti OR 'mobile application':ab,ti OR app:ab,ti OR 'm health':ab,ti OR mhealth:ab,ti) AND (obesity:ab,ti OR overweight:ab,ti OR 'body weight':ab,ti OR 'weight loss':ab,ti OR 'weight reduction':ab,ti OR 'weight management':ab,ti) AND (randomized:ab,ti OR randomised:ab,ti OR randomly:ab,ti OR random:ab,ti OR 'control group':ab,ti) | 526 |
| *The Cochrane Library (trials only)* | smartphone application OR phone application OR mobile application OR app OR m-health OR mhealth in Title Abstract Keyword AND Obesity OR overweight OR body weight OR weight loss OR weight reduction or weight management in Title Abstract Keyword AND Randomized OR randomised OR randomly OR random OR control group in Title Abstract Keyword - (Word variations have been searched) | 1,461 |
| *CINAHL* | AB ( smartphone application OR phone application OR mobile application OR app OR m-health OR mhealth ) AND AB ( Obesity OR overweight OR body weight OR weight loss OR weight reduction or weight management ) AND AB ( Randomized OR randomised OR randomly OR random OR control group ) | 188 |
| *PsycInfo* | ((smartphone application or phone application or mobile application or app or m-health or mhealth) and (Obesity or overweight or body weight or weight loss or weight reduction or weight management) and (Randomized or randomised or randomly or random or control group)).ab. | 76 |
| *Scopus* | ( TITLE-ABS-KEY ( smartphone AND application OR phone AND application OR mobile AND application OR app OR m-health OR mhealth ) AND ALL ( obesity OR overweight OR body AND weight OR weight AND loss OR weight AND reduction OR weight AND management ) AND TITLE-ABS-KEY ( randomized OR randomised OR randomly OR random OR control AND group ) ) | 236 |
| *Web of Science* | smartphone application OR phone application OR mobile application OR app OR m-health OR mhealth (Abstract) and Obesity OR overweight OR body weight OR weight loss OR weight reduction or weight management (Abstract) and Randomized OR randomised OR randomly OR random OR control group (Abstract) | 581 |
