# Supplementary material for: Sustainability of Weight Loss Through Smartphone Apps: Systematic Review and Meta-analysis on Anthropometric, Metabolic, and Dietary Outcomes
Source: J Med Internet Res. 2022 Sep 21;24(9):e40141. doi: 10.2196/40141 (PMC9536524; doi:10.2196/40141)
Supplement: Multimedia Appendix 3 [file jmir_v24i9e40141_app3.docx]

**APPENDIX 3** Extended study characteristics of the 16 included articles.

| **Author, year** | **SES, % (n)** | **Educational level, % (n)** | **Presence of group differences between retained and dropped out** | **Protocol registered?** | **Grant funded?** |
| --- | --- | --- | --- | --- | --- |
| Carter et al., 2013 | Occupation (Managerial and professional occupations) Smartphone: 74.4% Diary: 51.2% | Has a university degree Smartphone: 72.1% Diary: 55.8% | Weight (p=0.03), BMI (p=0.001), body fat (p=0.01), obese (p=0.05), reported health status as excellent or good (p=0.001) | Yes ClinicalTrials.gov NCT01744535 | Yes National Prevention Research Initiative grant (grant number G0802108) |
| Duncan et al., 2020 | Employment (4 categories- displaying only Full Time) Pooled Intervention: 53.8% Control: 52.8% Total: 52.4% | Years in Education Pooled Intervention: 17.1 (2.9) Control: 14.8 (2.7) Total: 16.4 (3.0) | Completers reported fewer symptoms for DASS-Depression (p<0.001) and DASS-Stress (p=0.036) | Yes ACTRN12617000735358; Universal Trial Number U1111-1219-2050) | Yes |
| Dunn et al., 2019 | Occupation Level No current employment: 4.7% Service occupation: 2.3% Technical, sales, administrative: 11.6% Executive, managerial 11.6% Professional specialty: 39.5% Retired: 2.3% Other: 30% | High school: 2.3 (1) Some college: 18.6 (8) College graduate: 30.3 (13)  Advanced degree: 48.8 (21) | NS | Yes NCT02868853 | Yes funded by the Academy of Nutrition and Dietetics Foundation Amy Joye Memorial Research Award |
| Eisenhauer et al., 2021 | Under $20,000: 1.3 (1) $20,000-$39,000: 7.7 (6) $40,000-$59,000: 9 (7) $60,000-$79,000: 20.5 (16) $80,000-$99,000: 24.4 (19) $100,000 or more: 37.2 (29) | High school graduate/GED: 7.5 (6) Some college but no degree: 16.3 (13) Associates degree: 17.5 (14) Bachelor's degree: 30 (24) Master's degree: 22.5 (18) Doctoral degree: 6.3 (5) | NS | Yes ClinicalTrials NCT03329079 | Yes NIH/NINR Health Disparities Section, 1R15NR017522–01 |
| Falkenhain et al., 2021 | $0 - $25,000: 18 (12) $25,000 - $50,000: 18 (12) $50,000 - $75,000: 19 (12) $75,000 - $100,000: 16 (10) $100,000 - $125,000: 18 (12) $125,000 - $150,000: 9 (6) $150,000 - $175,000: 8 (5) $175,000 +: 28 (18) Not specified: 21 (13) | Ketogenic Diet App, n(%): Apprenticeship or trades certificate or diploma: 7(9) College certificate or diploma: 11(14) High school graduate: 6 (8) Less than high school: 1(1) Post-graduate degree: 24 (31) University certificate, diploma or degree: 26 (34) Not specified: 2 (3)  Calorie-Restricted Low-Fat Diet App, n(%): Apprenticeship or trades certificate or diploma: 3(4) College certificate or diploma: 15 (19)  High school graduate: 7 (9) Less than high school: 2 (3) Post-graduate degree: 26 (33) University certificate, diploma or degree: 22 (28) Not specified: 3 (4)  Total, n(%): Apprenticeship or trades certificate or diploma: 10 (7) College certificate or diploma: 26 (17) High school graduate: 13 (8) Less than high school: 3 (2) Post-graduate degree: 50 (32) University certificate, diploma or degree: 48 (31) Not specified: 5 (3) | Nil | Yes ClinicalTrials.gov (NCT04165707) | Yes Supported by Canadian Institutes of Health Research (MSH141980), Michael Smith Foundation for Health Research (MSFHR 16890), and Mitacs (IT15608) |
| Godino et al., 2016 | (no suitable proxies given by study; all participants are college students) | (no suitable proxies given by study; all participants are college students) | Nil | Yes ClinicalTrials.gov (NCT01200459) | Yes The National Heart, Lung, and Blood Institute of the National Institutes of Health (U01 HL096715) |
| Johnston et al., 2013 | 64% employed for wages (Specific breakdown not stated in study) | 76.7% have at least some college credit (Specific breakdown not stated in study) | Nil | No | Yes # grant from Weight Watchers International, Inc |
| Kurtzman et al., 2018 | Gamification with PCP data sharing Less than $50,000: 21.9 (14) $50,000 to $100,000: 29.7 (19) Greater than $100,000: 37.5 (24) Unknown: 10.9 (7) Gamification Less than $50,000: 21.2 (14) $50,000 to $100,000: 34.8 (23) Greater than $100,000: 40.9 (27) Unknown: (3) (2) Control Less than $50,000: 19.7 (13) $50,000 to $100,000: 33.3 (22) Greater than $100,000: 34.8 (23) Unknown: 12.1 (8) | Gamification with PCP data sharing Less than college graduate: 28.1 (18) College graduate: 71.9 (46) Gamification Less than college graduate: 30.3 (20) College graduate: 69.7 (46) Control Less than college graduate: 31.8 (21) College graduate: 68.2 (45) | NS | Yes clinicaltrials.gov Identifier: 02564445 | Yes grants from the University of Pennsylvania Center for Therapeutic Effectiveness Research (CTER) and the McCabe Fund |
| Martin et al., 2015 | NS | NS | NS | Yes ClinicalTrials.gov identifier NCT00883350 | Yes National Institutes of Health (NIDDK) grants R03 DK083533A and P30 DK072476 |
| Patel et al., 2019 | Simultaneous:  $0-$49,999: 25 (8) $50,000-$99,999: 44 (14) $100,000 or greater: 28 (9) Unknown/not reported: 3 (1) Sequential:  $0-$49,999: 27 (9) $50,000-$99,999: 35 (12) $100,000 or greater: 32 (11) Unknown/not reported: 6 (2) App-Only: $0-$49,999: 27 (9) $50,000-$99,999: 29 (10) $100,000 or greater: 41 (14)  Unknown/not reported: 3 (1)  Total:  $0-$49,999: 26.0 (26)  $50,000-$99,999: 36.0 (36)  $100,000 or greater: 34.0 (34)  Unknown/not reported: 4.0 (4) | Simultaneous:  Less than college graduate: 22 (7) College graduate or above: 78 (25) Sequential:  Less than college graduate: 18 (6) College graduate or above: 82 (28) App-Only:  Less than college graduate: 12 (4) College graduate or above: 88 (30) Total: Less than college graduate: 17.0 (17) College graduate or above: 83.0 (83) | NS | Yes ClinicalTrials.gov NCT03254953 | Yes Grants to the first author from the American Psychological Association, the Duke Interdisciplinary Behavioral Research Center and the Aleane Webb Dissertation Research Award provided by The Graduate School at Duke University |
| Rosas et al., 2020 | Intervention:  <$75 000: 48.1 (39) $75 000 to <$125 000: 22.2 (18)  ≥$125 000: 29.6 (24) Usual Care:  <$75 000: 41.5 (34) $75 000 to <$125 000: 26.8 (22) ≥$125 000: 31.7 (26) Overall:  <$75 000: 44.8 (73) $75 000 to <$125 000: 24.5 (40)  ≥$125 000: 30.7 (50) | Intervention:  ≤High school or GED: 28.9 (26) Some college: 24.4 (22) College graduate: 23.3 (21) Post college: 23.3 (21) Usual Care:  ≤High school or GED: 28.9 (28) Some college: 28.9 (28) College graduate: 20.6 (20)  Post college: 21.7 (21) Overall: ≤High school or GED: 28.9 (54) Some college: 26.7 (50) College graduate: 21.9 (41) Post college: 22.5 (42) | NS | Yes ClinicalTrials.gov Identifier: NCT02459691 | Yes Supported by the Agency for Healthcare Research and Quality under award No. R01HS022702 |
| Ross et al., 2016 | NS | NS | NS | ClinicalTrials.gov identifier NCT01999244 | Yes Support for this study provided by the National Institute of Diabetes and Digestive and Kidney Diseases (National Institutes of Health) under award number F32 DK100069 awarded to first author |
| Spring et al., 2017 | NS | College graduate or above: TECH: 68.8 STND: 65.6 SELF: 71.9 Total: 68.8 | Attrition for SELF is greater than either STND or TECH (p=0.02), but not differential between STND and TECH (p=0.20) # Study did not specify difference between the general retained sample vs. lost to follow-up sample | ClinicalTrials.gov identifier NCT01051713 | Yes  Supported in part by grants RC1DK087126 and R01DK097364 from the National Institute of Diabetes and Digestive and Kidney Diseases and by the Robert Lurie Comprehensive Cancer Center Support Grant (P30CA60553) and the Northwestern University Clinical Translational Science Award (UL1TR001422) |
| Tanaka et al., 2018 | NS | NS | NS | Yes University Hospital Medical Information Network (UMIN) Clinical Trials Registry (UMIN000025340) | Yes  financially supported by a collaborative research agreement between Faculty of Health and Sport Sciences at the University of Tsukuba and FiNC Inc, and a research contract between FiNC Inc and THF Co., Ltd. Genki Plaza Medical Center for Health Care |
| Turner-McGrievy et al., 2017 | App: No current employment: 4.8 (2) Service occupation: 11.9 (5) Technical, sales, administrative: 9.5 (4) Executive, managerial: 4.8 (2) Professional specialty: 40.5 (17) Retired: 9.5 (4) Other: 19 (8) Bite: No current employment: 2.6 (1) Service occupation: 10.3 (4) Technical, sales, administrative: 23.1 (9) Executive, managerial: 7.7 (3) Professional specialty: 17.9 (7) Retired: 5.1 (2) Other: 33.3 (13) | App: High school or some college: 11.9 (5) College graduate: 42.9 (18) Advanced degree: 45.2 (19) Bite: High school or some college: 18 (7) College graduate: 48.7 (19) Advanced degree: 33.3 (13) | Nil | ClinicalTrials.gov identifier NCT02632461 | Yes funded by the National Cancer Institute of the National Institutes of Health under award number R21CA18792901A1 |
| Zhou et al., 2021 | NS | Dietary and physical activity interventions group (DPG):  Elementary school or below: 18.4 (46) Middle or high school: 61.2 (153) University studies: 20.4 (51) Physical activity interventions group (PG): Elementary school or below: 18.5 (46) Middle or high school: 60.5 (150) University studies: 21.0 (52) Control group (CG):  Elementary school or below: 19.4 (48) Middle or high school: 61.7 (153) University studies: 19.0 (47) Total:  Elementary school or below: 18.8 (140) Middle or high school: 61.1 (456) University studies: 20.1 (150) | NS | Yes Chinese Clinical Trial Registry (ChiCTR1900023355) | Yes Yingdong Intelligent Technology (Shandong) Co., Ltd. Provivided fund for the project |
