# Supplementary material for: Sustainability of Weight Loss Through Smartphone Apps: Systematic Review and Meta-analysis on Anthropometric, Metabolic, and Dietary Outcomes
Source: J Med Internet Res. 2022 Sep 21;24(9):e40141. doi: 10.2196/40141 (PMC9536524; doi:10.2196/40141)
Supplement: Multimedia Appendix 4 [file jmir_v24i9e40141_app4.docx]

**APPENDIX 4:** Intervention characteristics of the 16 included articles.

| **Author, year** | **App components** | **Control condition / Intervention duration / Follow-up timepoints** | **Key findings** |
| --- | --- | --- | --- |
| Carter et al., 2013 | Self-monitoring of diet, physical activity and weight with tailored weekly text messages triggered according to users' progress toward their calorie targets | Diary: Calorie-counting book / 6 months / Baseline, 6 weeks, 6 months | - Adherence significantly higher in smartphone group than diary group - Self-monitoring declined over time in all group - BMI change at 6 months: Smartphone = -1.6kg/m^2^, Diary = -1.0kg/m^2^ - Change in body fat: Smartphone = -1.3%, Diary = -0.9% |
| Duncan et al., 2020 | Balanced Smartphone App: education, goal setting, self-monitoring of weight, diet and exercise, feedback  EXTRA components: Calorie counting platform, body weight scale, Fitbit activity tracker, participant handbook | Wait-list control / 12 months / Baseline, 6 months, 12 months | - No significant group differences in weight between at 6 and 12 months - Pooled Intervention group significantly increased resistance training and reduced energy intake at 6 months and improved insomnia symptoms at 12 months |
| Dunn et al., 2019 | FatSecret app (Self-monitoring of diet)- users can enter the amounts and types of foods and beverages consumed selected from a database or manually entered | Photo Group: Meal-Logger app: photo food journaling; rate and view self and other users' foods, track their diet using Traffic Light Diet / 6 months / Baseline, 6 weeks, 6 months | - No significant group differences in weight outcomes but both groups achieved significant weight loss. - Overall low adherence of food logging (<30% of days) - Food logging was significantly correlated with weight change for calorie tracking group but not the photo tracking group |
| Eisenhauer et al., 2021 | Lose-It! app  Premium version (MT+): Real-time self-monitoring of diet and exercise, enhanced customisation of personalised reports, discussion forum, sync weight from smart scale, feedback regarding weight trends | Basic version of Lose-It! App: Real-time self-monitoring via manual logging / 6 months / Baseline, 3 months, 6 months | - At 6 months, observed mean weight loss was 7.03 kg for MT+ group and 4.14 kg for MT group, with 42.9% and 34.2% meeting ≥5% weight loss, respectively |
| Falkenhain et al., 2021 | Ketogenic diet app (Paired with breath acetone biofeedback device): traffic light system to recommend consumption of foods according to amount of net carbohydrates; users would measure their acetone level 3 times daily and receive feedback to manage carbohydrate intake | Calorie-restricted low-fat diet app: points-based food tracking / 12 weeks / Baseline, 12 weeks, 24 weeks | - Significantly higher weight loss in ketogenic group at 12 and 24 weeks. - Greater improvement in hemoglobin A1c and liver enzymes in ketogenic diet group |
| Godino et al., 2016 | GoalGetter App: Set weight-related goals and review progress ad hoc; information can be shared with others BeHealthy App: Used to deliver 2 weight-related challenges per day; information can be shared with others TrendSetter App: Self-monitor weight, physical activity and diet daily; graphs of trends over time can be viewed and shared | Quarterly newsletters via email on health topics relevant to young adults Website with general weight loss information / 24 months / Baseline, 6 months, 12 months, 18 months, 24 months | - Significantly higher weight loss in intervention group at 6 and 12 months - No significant group differences at 18 and 24 months |
| Johnston et al., 2013 | Weight Watchers eTool: Access food, activity and weight-monitoring systems; library of meal ideas, recipes and content on a variety of weight-related topics; seek group support through participating in community message and discussion boards | Provided publicly available printed materials on basic dietary and exercise guidelines for safe weight loss; public library materials, web sites, telephone numbers of health promotion organisations offering free weight control information / 6 months / Baseline, 3 months, 6 months | - Weight Watchers Group participants significantly decreased their body mass index at 6 months (F = 36.7, P <.001) and were 8.0 and 8.8 times more likely to achieve a 5% and 10% reduction in weight, respectively, compared with those in the self-help group - High usage of all 3 access modes (meetings, Weight Watchers website, mobile device application) resulted in the greatest weight loss (P <.001) |
| Kurtzman et al., 2018 | HealthMate app: Tracking daily step counts  Participant were also given a wireless weight scale | No treatment / 24 weeks / Baseline, 12 weeks, 24 weeks, 36 weeks | - Significant weight loss in all three arms at 24 and 36 weeks - No significant group differences in weight loss between each of the intervention arms and control at either 12, 24, or 36 weeks |
| Martin et al., 2015 | SmartLoss: Remote monitoring of progress and delivery of personalised treatment recommendations and lesson materials; guidance on gradually increasing physical activity | Received health information via text messages or emails delivered to the smartphones / 12 weeks / Basline, Week 4, Week 8, Week 12 | Significantly higher weight loss in SmartLoss group at weeks 4, 8 and 12. |
| Patel et al., 2019 | MyFitnessPal app: tracking of body weight and dietary intake daily with behaviour change techniques | Tracking of diet daily with MyFitnessPal app but did not receive additional behaviour change techniques / 12 weeks / Baseline, 1 month, 3 months | - No significant group difference in weight change at 3 months. - The median number of days of self-monitoring diet per week was 1.9 (interquartile range [IQR] 0.3-5.5) in Sequential (once began), 5.3 (IQR 1.8-6.7) in Simultaneous, and 2.9 (IQR 1.2-5.2) in App-Only |
| Rosas et al., 2020 | 2 apps: (unspecified application): Track physical activity MyFitnessPal: Track dietary intake  Other components include group sessions, individualised feedback from health coach, monthly emails | Usual care from primary care clinicians; not prevented from accessing weight management services / 24 months / Baseline, 12 months, 24 months | - Significantly higher weight loss in intervention group at 12 months. - No significant group difference in weight change at 24 months. - significantly greater in the intervention group (−2.6 [6.0] kg) than the control group (−0.3 [4.2] kg) at 12 months (mean difference, −2.1 [95% CI, −3.6 to −0.7] kg; P = .005)  Intervention participants were more likely to achieve at least 5%weight loss than control participants at 12 months (22 participants [25.9%] vs 9 participants [9.2%]; P = .003), and participants who achieved at least 5%weight loss attended more intervention sessions than those who did not (mean [SD], 16.6 [7.6] sessions vs 12.4 [7.5] sessions; P = .03). |
| Ross et al., 2016 | Fitbit app: Track caloric intake + app provided updates on calorie target # Participants also given a step tracker and Fitbit Aria scale that sync with the app, letting participants view the graphs of their caloric intake, physical activity, and weight over time | Provided with self-monitoring tools: Calorie reference book, pedometer to monitor daily step counts, body weight scale (if participants don't have one) / 6 months / Baseline, 3 months, 6 months | - TECH+PHONE (-6.4±1.2 kg) lost significantly more weight than ST (-1.3±1.2 kg); weight loss in TECH (-4.1±1.4 kg) was between ST and TECH+PHONE - Fewer ST (15%) achieved ≥5% weight losses compared with TECH and TECH+PHONE (44%), P=0.039 - Adherence to self-monitoring caloric intake was higher in TECH+PHONE than TECH or ST, Ps < 0.05 |
| Spring et al., 2017 | ENGAGED app (study-designed): Self-monitor dietary intake and body weight; has a team tab to track their group members' self-monitoring adherence, post messages to individuals or teams # Participants are also given an accelerometer to measure MVPA; data is sent to a coach and ENGAGED app, where participants can visualise the remaining MVPA needed | SELF group (control): Diabetes Prevention Programme as a self-guided programme on DVD; paper self-monitoring diaries STND (Standard) group: Paper and pencil self-monitoring (but receive DPP from an app like TECH [intervention] group) / 6 months (follow-up at 12 months) / Baseline, 3 months, 6 months, 12 months | - Weight loss significantly greater for TECH and STND than SELF at 6 months but not 12 months. - TECH and STND did not differ except that more STND (59%) than TECH (34%) achieved5% weight loss at 6 months (P<0.05). - Self-monitoring adherence was greater in TECH than STND (P<0.001), greater in both interventions than SELF (P<0.001), and covaried with weight loss (r(84)50.36-0.51, P<0.001). |
| Tanaka et al., 2018 | Self-monitor weights and meals: Measure body weight twice a day, take photos of their meals and upload to a group chat for users; nutrition professionals give participants feedback on their meals as well as to answer questions; communicate with users in the same grouping (participants assigned to a group of up to 6 individuals) | No treatment / 8 weeks (+ 4 weeks of follow-up = 12 weeks) / Baseline, Week 8, Week 12 | - Significantly larger 8-week weight loss in the coaching group - Significantly larger improvements in triglyceride and glycated hemoglobin A1c levels were also obtained in the coaching group. - Weight loss and glycated hemoglobin A1c improvement maintained until week 12 - The frequent upload of meal photos was associated with a larger 8-week weight loss in a dose–response fashion (P-value for trend <0.001) |
| Turner-McGrievy et al., 2017 | FatSecret app: Record all food and beverages consumed each day to track total daily energy intake Participants were also given a list of free pedometer app or given a pedometer: Self-monitor their exercise by tracking steps | BITE group: Bite Counter device that looks like a watch: Monitor intake by counting bites using a microelectromechanical gyroscope / 6 months / Baseline, Month 3, Month 6 | - App group lost significantly more weight than the Bite group - Changes in energy intake (kcal/d) or number of days diet did not differ between groups, but the Bite group had significant increases in physical activity metabolic equivalents (12015.46684.6 min/wk; P50.02) compared to little change in the App group (2136.56630.6; P50.02). - Total weight loss was significantly correlated with number of podcasts downloaded (r520.33, P<0.01) and number of days diet was tracked (r520.33, P<0.01) |
| Zhou et al., 2021 | Mobile-based support system: Connect users with dieticians or sports coaches; syncs data from sports bracelet and weighing scale and display graphic feedback and recommendations; users can also upload food logs and pictures of their daily meals with standardised meal plates. Participants were also given sports bracelets and weighing scale | No treatment / 3 months / Baseline, 45 days, 90 days | - Comparing with PG and CG, the DPG showed a significant decrease in all outcomes after three months, including body weight, BMI, and HC. Similar effects were seen across sex and BMI subgroups. |
