# Supplementary material for: Sustainability of Weight Loss Through Smartphone Apps: Systematic Review and Meta-analysis on Anthropometric, Metabolic, and Dietary Outcomes
Source: J Med Internet Res. 2022 Sep 21;24(9):e40141. doi: 10.2196/40141 (PMC9536524; doi:10.2196/40141)
Supplement: Multimedia Appendix 5 [file jmir_v24i9e40141_app5.docx]

**APPENDIX 5**: Methodological quality assessment of 16 included articles using the Cochrane Risk of Bias tool.

| Study ID | Random | allocation | Performance | Detection | Attrition | Reporting | Overall |
| --- | --- | --- | --- | --- | --- | --- | --- |
| Carter et al., 2013 | L | U | H | U | H | L | H |
| Duncan et al., 2020 | L | U | H | L | L | L | H |
| Dunn et al., 2019 | L | U | U | L | L | L | U |
| Eisenhauer et al., 2021 | L | U | H | H | L | L | H |
| Falkenhain et al., 2021 | L | U | L | L | L | L | U |
| Godino et al., 2016 | L | L | H | L | L | L | H |
| Johnston et al., 2013 | L | U | U | U | L | L | U |
| Kurtzman et al., 2018 | L | U | U | L | L | L | U |
| Martin et al., 2015 | L | U | U | L | L | L | U |
| Patel et al., 2019 | L | H | H | H | L | L | H |
| Rosas et al., 2020 | L | U | L | L | L | L | U |
| Ross et al., 2016 | L | U | U | L | L | L | U |
| Spring et al., 2017 | L | U | U | L | L | L | U |
| Tanaka et al., 2018 | L | L | U | L | L | L | U |
| Turner-McGrievy et al., 2017 | L | U | U | L | L | L | U |
| Zhou et al., 2021 | L | U | H | L | H | L | H |

Note: L=low risk of bias; U=unclear risk of bias; H= high risk of bias
