# Supplementary material for: Sustainability of Weight Loss Through Smartphone Apps: Systematic Review and Meta-analysis on Anthropometric, Metabolic, and Dietary Outcomes
Source: J Med Internet Res. 2022 Sep 21;24(9):e40141. doi: 10.2196/40141 (PMC9536524; doi:10.2196/40141)
Supplement: Multimedia Appendix 6 [file jmir_v24i9e40141_app6.docx]

**APPENDIX 6** Summary of the outcomes reported in each study.

| **Author, year** | **Weight (kg)** | **Waist circumference** | **Calorie intake** | **HDL-C** | **LDL-C** | **HbA1c** | **Blood pressure** |
| --- | --- | --- | --- | --- | --- | --- | --- |
| Carter et al., 2013 | - 6 weeks: sig - 6 months: sig | x | x | x | x | x | x |
| Duncan et al., 2020 | - 6 months: no - 12 months: no | - 6 months: sig - 12 months: no sig | - 6 months: sig - 12 months: no | x | x | - 6 months: no - 12 months: no | x |
| Dunn et al., 2019 | - 6 weeks: no - 6 months: no | x | x | x | x | x | x |
| Eisenhauer et al., 2021 | - 3 months: no - 6 months: sig | x | x | x | x | x | x |
| Falkenhain et al., 2021 | - 3 months: sig - 6 months: sig | x | - 3 months: sig - 6 months: sig | - 3 months: no - 6 months: no | - 3 months: no - 6 months: no | - 3 months: sig - 6 months: sig | x |
| Godino et al., 2016 | - 6 months: no - 12 months: sig - 18 months: sig - 24 months: sig | - 6 months: sig - 12 months: no - 18 months: no - 24 months: no | x | x | x | x | Systolic   - 6 months: no - 12 months:no - 18 months:no - 24 months:sig   Diastolic   - 6 months: no - 12 months: no - 18 months:no - 24 months: no |
| Johnston et al., 2013 | - 3 months: sig - 6 months: sig | x | x | x | x | x | x |
| Kurtzman et al., 2018 | - 3 months: no - 6 months: no - 9 months: no | x | x | x | x | x | x |
| Martin et al., 2015 | - 1 month: sig - 2 months: sig - 3 months: sig | - 1 month: sig - 2 months: sig - 3 months: sig |  |  |  |  | - 1 month: no - 2 months: no - 3 months: no |
| Patel et al., 2019 | - 1 month: no - 3 months: no - 6 months: no | x | x | x | x | x | x |
| Rosas et al., 2020 | - 12 months: sig - 24 months: no | x | - 12 months: no - 24 months: no | x | x | x | x |
| Ross et al., 2016 | - 3 months: sig - 6 months: sig | x | x | x | x | x | x |
| Spring et al., 2017 | - 3 months: sig - 6 months: sig - 12 months: no | x | x | x | x | x | x |
| Tanaka et al., 2018 | - 2 months: sig - 3 months: sig | - 2 months: no - 3 months: sig | x | - 2 months: no - 3 months: no | - 2 months: no - 3 months: no | - 2 months: sig - 3 months: sig | Systolic   - 2 months: no - 3 months: no   Diastolic   - 2 months: no - 3 months: no |
| Turner-McGrievy et al., 2017 | - 3 months: no - 6 months: sig | x | - 3 months: no - 6 months: no | x | x | x | x |
| Zhou et al., 2021 | - 45 days: sig - 3 months: sig | - 45 days: sig - 3 months: sig | x | x | x | x | x |

Note: High-density lipoprotein cholesterol (HDL-C); Low-density lipoprotein cholesterol (LDL-C); sig=significant differences between the intervention and control groups; no=no significant differences between the intervention and control groups
